# Measuring the Empowerment of International Organizations: The Evolution of Financial and Staff Capabilities

**DOI:** 10.1111/1758-5899.12449

**Published:** 2017-08-24

**Authors:** Eugénia Heldt, Henning Schmidtke

**Affiliations:** ^1^ Bavarian School of Public Policy TUM School of Governance Technical University of Munich

## Abstract

International organizations’ (IOs) power in shaping global governance outcomes is not only determined by the formal delegation of tasks and issue areas but also by the necessary capabilities to fulfill these tasks. Yet, extant research on the delegation of power to IOs gives few insights into the financial and staff capabilities of IOs and focuses mainly on the formal rules that specify IOs’ tasks and issue scope. To address these limitations, this paper makes three contributions. First, we propose a more encompassing concept of IO power which incorporates three principal components: tasks, issue scope, and capabilities. Second, we introduce a new concept – IO empowerment (IOE) – which encapsulates formal and informal changes in IO power over time. Third, we introduce a novel dataset on IO capabilities, which measures the formal rules governing IO staff and financial resources as well as the actual capabilities available to six well‐known IOs over 65 years. These original data show that capabilities vary not only across IOs but also over time.

**Policy Implications**

- The power of international organizations has three principal components: tasks; issue scope; and capabilities.
- When the number of issue areas delegated to IOs grows and when the type of issue areas become more intrusive, IO power increases.
- The more financial resources are available to an IO, the higher the organization's power.
- IOs may strategically use financial and human resources at their disposal to empower themselves over time.

The growing importance of international organizations (IOs) has caused scholars to focus their attention on how power is delegated to IOs and what shapes IOs’ power to affect global governance outcomes. We have learned quite a bit about *why* states decide to delegate power to IOs (Abbott and Snidal, 1998; Hawkins et al., 2006; Pollack, 2003), *how* states choose institutional designs (Bradley and Kelley, 2008; Jupille et al., 2013; Koremenos et al., 2001), *which control mechanisms* mediate principal‐agent (henceforth, PA) relationships (Graham, 2014; Grigorescu, 2010; Johnson, 2014), and *what consequences* result from transferring tasks to IOs (Conceição‐Heldt, 2010; Tallberg, 2002; Zürn et al., 2012). The respective empirical analyses of IOs’ formal institutional designs (Hooghe and Marks, 2015; Lipscy, 2015) have contributed to a growing understanding of what shapes IOs’ ability to affect global governance outcomes.

In contrast, the capabilities of IOs to perform the tasks formally delegated to them in different issue areas – the focus of this special issue and our contribution – have received considerably less attention (Goetz and Patz, 2017). Capabilities refer in this piece to the financial and human resources available to an IO. Many International Relations (IR) scholars simply assume that IO financial resources and staff are limited and that they are subject to close member state oversight (see, for instance, Abbott et al., 2015; Abbott and Snidal, 1998; Barnett and Finnemore, 2004). This broad assumption is striking because IOs’ power to shape global governance outcomes is clearly contingent on their financial and staff capabilities (Brown, 2010; Graham, 2015; Parízek, 2016). As Amerasinghe (2005, p. 352) argues: ‘Financing is at the heart of the functioning of international organizations. Without adequate funds they could not achieve their purposes and functions’. Along these lines, Eckhardt and Dijkstra (2017) demonstrate how IO budgets shape the implementation of international policies; Abbott et al. (2015) show that IOs’ material resources affect the extent to which they cooperate with intermediary actors to achieve their governance targets; Graham (2017) highlights how different methods of funding shape the nature of IO governance; Ege and Bauer (2017) investigate how different financial sources affect IO autonomy; and Squatrito (2017) examines the resource management design of international courts. Similarly, research focusing on IOs as bureaucracies (Trondal et al., 2010) has demonstrated how international bureaucrats shape IOs’ institutional design (Johnson, 2014), behavior (Barnett and Finnemore, 2004), and policy output (Weaver, 2008). This research indicates that IO staff has a significant impact on how IOs shape global governance.

Against this background, this article seeks to integrate the conceptual and empirical literature on formal delegation with more recent scholarship on IO capabilities. In so doing, we make three contributions. First, we suggest that IO power can be understood as an organizational outcome with three principal components: *tasks*,* issue scope*, and *capabilities*. While the literature on delegation focuses mainly on formally delegated tasks and the scope of issue areas in which these tasks are performed, we view financial and staff capabilities as a third main component of IO power. An IO is powerful when it performs a broad set of tasks, such as agenda setting, dispute settlement, or fund distribution, carries out these tasks in a broad array of domestically intrusive issue areas *and* possesses the necessary financial and staff capabilities to perform these tasks. Capabilities are not only an important element of IO power, they are also a key prerequisite for high‐quality performance. Bureaucracies with strong independent capabilities are more likely to perform their functions well (Evans, 1995, Fukuyama, 2013).

Second, we introduce the concept of IO empowerment (henceforth, IOE), which depicts the delegation of power to IOs as a process evolving over time. This concept allows us to better integrate temporal and dynamic aspects into the analysis of power delegation to IOs. IOE refers to the organizational processes that shape the tasks, scope, and capabilities of IOs over time. These processes do not only take the form of formal institutional change, they also come in more subtle, incremental modes which nevertheless have the potential to substantively shape IOs’ power over time (see also Rixen et al., 2016).

Third, we illustrate the empirical plausibility and relevance of our concepts using a novel dataset on IO capabilities. Some notable exceptions notwithstanding (Brown, 2010; Graham, 2015; Gray, 2016; Michaelowa and Michaelowa, 2017; Vaubel et al., 2007), systematic comparative and longitudinal empirical data on IO budgets and staff are few and far between. To address this gap, our dataset covers the capabilities of six well‐known IOs: the European Union (EU), the General Agreement on Tariffs and Trade/World Trade Organization (GATT/WTO), the International Monetary Fund (IMF), the World Health Organization (WHO), the United Nations Educational, Scientific and Cultural Organization (UNESCO), and the World Bank Group (WBG).

We proceed as follows. First, we introduce the concepts of IO power and empowerment. For this purpose, we turn to the IR literature on power, the PA literature on formal delegation, and organization theory. Second, we outline the operationalization of IO capabilities as one principal component of IO power. Third, we present the dataset on IO capabilities and map the evolution of the selected IOs’ staff and financial resources since their establishment. Finally, we summarize our findings and suggest research strategies for explaining the variance of IO power across organizations and over time.

## Conceptualizing IOE

When is an IO powerful and what makes it a powerful actor in global governance? Barnett and Finnemore (2004, p. 7) postulate that IOs are powerful actors in today's global governance and Stone (2011, p. 1) asserts that the IMF is among the most powerful IOs. Yet, what does this mean concretely? How can we conceptualize the power of IOs?

Conceptually and theoretically, the organizational outcome of IO power and its constitutive processes of empowerment are related to terms such as delegation, agency, and authority. Yet, the link between the formal delegation of power and IOs’ actual power to shape global governance outcomes has remained unclear due to a limited focus on IO capabilities. In the following section, we argue that IO power has three principal components: the number and importance of the tasks delegated to them by states, the scope and intrusiveness of issue areas in which IOs perform these tasks, and the staff and financial capabilities that underpin this work. In the subsequent section, we turn to the two types of processes which drive the dynamics of IO power over time: formal and informal IOE. While much of the present literature focuses on formal changes to IO mandates, we propose that IO power may change incrementally even if IOs’ formal rules remain unaltered.

### Principal components of IO power

Traditionally, IR scholars use the concept of power, which is a central part of realist thinking, when referring to states, not to IOs (Waltz, 1979). A state is assumed to be powerful if it disposes of, for instance, economic resources, such as high domestic production, or if it has the military capabilities to affect others and obtain the outcomes the state wants (Baldwin 2002). This notion of power highlights the ability of state A to change the behavior of state B by coercive means or through financial incentives. Other authors less supportive of the realist focus on financial and military capabilities have offered more encompassing concepts of power. Nye (1990, p. 166) argues, for instance, that hard forms of compulsory power are complemented by soft forms of power, which occur when one state is able to get another actor ‘to want what it wants’. Like Barnett and Duvall (2005), Nye claims that soft power is shaped not so much by hard capabilities as by cultural and ideological attraction or the rules and institutions of international regimes. These diverse concepts of power share a basic premise: to exercise power, actors must have preferences, seek to achieve those preferences, and have some margin of agency to apply different forms of power to reach their goals.

PA approaches and organization theory help us find an answer to what constitutes this agency for IOs. Most fundamentally, both strands of literature have demonstrated that IOs are best conceptualized as partially independent actors (Conceição‐Heldt et al., 2015). In contrast to realist approaches, which see IOs as mere *tools* of powerful states (Mearsheimer, 1994), and rational institutionalism, which conceptualizes IOs mainly as *fora* for international politics (Keohane, 1984), more recent research focusing on IOs as bureaucracies has highlighted their potential to shape global governance independently (Barnett and Finnemore, 2004). PA theory applied to IOs dissects situations where national governments (the collective principal) conditionally grant authority to an IO (the agent). By doing so, collective principals empower agents to perform specified tasks on their behalf (Koremenos, 2008; Lake, 2007, Pollack, 1997). IO secretariats are the key non‐state bodies to which tasks are delegated (Hooghe and Marks, 2015). The formal contract between the principal and the agent specifies the types of tasks the IO is asked to perform and the scope of issue areas in which these tasks shall be carried out (Hawkins et al., 2006). The literature aiming to measure the extent of this formal delegation has strongly focused on two principal components of IO power: tasks and issue scope.

The number and types of *tasks* delegated to an IO are a principal component of IOs’ institutional design (Koremenos et al., 2001) as well as being central to the concept of delegation examined in the PA literature. Examples of tasks performed by IOs include completing executive services, monitoring compliance, and distributing funds. Early attempts to measure this dimension of institutional design often blurred the distinction between the pooling of decision‐making authority by member states at an IO and the delegation of tasks to an international bureaucratic body (Lake, 2007). Boehmer et al. (2004), for instance, classified the institutionalization of 297 IOs as either minimal (lack of any bureaucratic, executive, or judicial organs that possess formalized power), structured (minimal state sovereignty is transferred to IO bodies), or interventionist (IOs possess mechanisms for coercing or influencing state behavior). Börzel (2005) described the centralization of tasks at the EU in more detail, combining decision‐making rules among member states (pooling) and the performance of agenda setting and judicial review tasks by an international administration (delegation). Similarly, Haftel and Thompson (2006) analyzed the independence of 30 regional IOs, on the basis of decision‐making rules, international bureaucracy, and third party dispute settlement. A more precise analysis has been provided by Hooghe and Marks (2015). In their study on the authority of 72 IOs, they distinguish clearly between pooling and delegation. Regarding delegation, the authors assess whether an IO is formally empowered to perform tasks in seven domains (executive functions, policy initiation, budget drafting, financial non‐compliance, member state accession, suspension of a member state, and constitutional revision). We follow this perspective, arguing that IOs become more powerful when more tasks are delegated to them and when tasks become more intrusive.

*Scope* refers to the issue areas in which IOs are allowed to operate (Koremenos et al., 2001). Here scholars must determine whether an IO was designed for narrow, policy specific issues, whether it operates more generally within an entire issue area, or whether it is a general‐purpose organization operating in a variety of issue areas. Following early attempts to classify IOs according to their scope (Jacobson et al., 1986; Shanks et al., 1996), a number of empirical studies applied broad schemes categorizing IOs as security, economic, social, or general‐purpose organizations (Boehmer et al., 2004; Volgy et al., 2008). More recent studies have expanded this approach and apply more detailed lists of issue areas. For example, Haftel (2013) looks at the scope of economic activity of regional economic IOs in 24 issue areas. Börzel (2005) codes 18 issue areas to analyze the issue scope of the EU and Hooghe and Marks (2015) use a list 25 issue areas to gauge the issue scope of 72 IOs. As with IO tasks, we argue that, when the number of issue areas delegated to IOs grows and when the type of issue area becomes more intrusive, IO power increases.

These two dimensions of formal institutional design are clearly at the core of IO power. They specify what IOs are expected to do and, thus, constitute an important element of IOs’ agency. Yet, the exclusive focus on these formal components comes at a significant cost. Doing so neglects the fact that exercising authority over a set of actors requires a bureaucratic staff that is sufficiently well equipped to execute general mandates and to implement specific policies (Weber, 1978, p. 212). As Trondal et al. (2010, p. 5) state, bureaucracies are ‘a key engine of international organizations’. Without them, IOs are barely more than a set of rules and procedures. To implement rules the staff of an IO must also have sufficient financial resources at its disposal. Hence, we propose a concept of IO power which views these capabilities as a third principal component of IO power.

*Capabilities* are the financial and human resources available to an IO (Brown, 2010). Regardless of which specific tasks are delegated to an IO and how broad the scope of their application is, the performance of any task by IOs necessitates personnel and financial resources. Without these basic resources, IOs cannot act. This is even the case if we assume a more constructivist perspective on IO power and different types of IOs. In the absence of resources, operative IOs cannot actively manipulate incentives to shape the behavior of other actors, nor can regulatory IOs hope to create, define, and map social reality to define problems, to identify legitimate means to pursue collective interests, or to establish the rules of a social situation.^1^ Both types of power and IOs require personnel and financial capabilities. The empirical part of this contribution focuses on this third principal component of IO power.

### Types of IOE

So far we have introduced the principal components of IO power. In the second step, we turn to the processes that drive these organizational outcomes. Most of the present literature on formal delegation assumes a comparative static perspective which misses the substantial changes in IO power taking place after the delegation contract has been signed. From the PA perspective, formal IOE takes place when member states explicitly delegate power to IOs by signing an agreement stipulating an agent's tasks, scope, and capabilities. Following the rational institutionalist premise that international institutions have to be sticky in order to fulfill their main function of stabilizing international cooperation (Abbott and Snidal, 1998, p. 10), these formal contracts are expected to remain unchanged over extended periods of time. Although longitudinal studies on institutional inertia (Shanks et al., 1996) are scarce, recent data indicate that many delegation contracts are indeed stable. In their study of formal delegation processes, Lenz et al. (2015, p. 147) show, for instance, that the number of delegated tasks has barely changed between, 1975 and, 2010 for many of the 72 IOs in their sample.

We argue that IOE is considerably more dynamic than the formal delegation perspective suggests. The tasks, scope, and capabilities of IOs change considerably over time for all IOs in our sample. Consequently, we propose the concept of IOE as a means of better assessing the temporal dynamics of power transfer from principals to agents over time. IOE refers to the *organizational process* of transferring specific tasks and resources to IOs over time. This process occurs, when new tasks are delegated to IOs, when tasks are extended to novel issue areas, and when staff and/or financial capabilities expand.^2^ The processes of IOE for all three components of IO power can be formal or informal.

*Formal IOE* refers to explicit, formal changes to IO founding treaties, rules of procedure, and other legal documents regulating the tasks to be performed by IOs and the resources available to IOs. Formal IOE, thus, takes place when member states explicitly decide to (re‐)regulate IO power in relation to one of the three principal power components by changing an IO's formal mandate. Historical institutionalists have labeled these processes displacement, that is, the removal of existing rules and the introduction of new ones, and layering, that is, the introduction of new rules on top of existing ones (Mahoney and Thelen, 2010, p. 15).

*Informal IOE*, by contrast, occurs when new tasks are added to an IO's portfolio, when the issue areas in which IO tasks are performed are extended, and when staff and financial capabilities increase without changing the formal delegation contract. In contrast to formal reform, informal IOE takes the form of more incremental and subtle changes in the interpretation and application of an IO's formal mandate both by member states and international bureaucrats (see also Hanrieder, 2014). These processes come either in the form of drift, defined as the changing impact of rules due to shifts in the environment, or through conversion, that is, a change in how existing rules are interpreted and implemented (Mahoney and Thelen, 2010, p. 16).

## Operationalizing IO capabilities

Since much has already been written about the measurement of IOs’ tasks and scope, the following section focuses on how to operationalize formal and informal IOE in relation to the third principal component of our concept of IO power, namely, capabilities. To capture formal and informal processes of IOE, we follow Brown's (2010) approach and examine both the formal rules governing IO decision‐making about staff and financial resources (*de jure* capabilities) and the *de facto* capabilities available to IOs at a given point in time.

In Table 1 we list four numerical indicators to measure de jure and de facto capabilities. The first set of indicators identifies and ranks formal decision rules on staff and financial capabilities (see also Squatrito, 2017). We propose that IOs in which decisions about staff and financial capabilities lie exclusively in the hands of member states are less powerful than those IOs where these decisions are made more independently of member states. We measure de jure staffing capabilities with the help of a four‐level scale.^3^ De jure staffing capabilities are weakest (‘1’) when there is a formal requirement for collective principal approval of staff appointments. In this case, staffing decisions are subject to close scrutiny by an oversight body. This prevents an IO from simply recruiting the staff it wants, forcing it instead to take into account each individual principal's preferences. De jure staffing capabilities are strongest (‘4’) when there is no requirement for principal approval of staff appointments.^4^

**Table 1:** Measuring IO capabilities

Indicator	Description	Coding Range
*De jure* capabilities		
Staffing	Requirement for collective principal approval of staff appointments for more than 2/3 of staff exists. Requirement for individual principal approval of staff appointments for more than 2/3 of staff exists. No requirement for individual principal approval of staff appointments for more than 2/3 of staff exists. No requirement for collective principal approval of staff appointments for more than 2/3 of staff exists.	0–4
Financing	+1: The agent is not solely funded by voluntary member state contributions. +1: The agent has access to obligatory member contributions. +1: The agent is allowed to charge fees for services. +1: The agent is allowed to accept voluntary contributions from non‐members or private actors. +1: The agent is allowed to levy taxes or collect similar mandatory contributions beyond member state contributions.	Additive index 0–5
*De facto* capabilities		
Staff	Number of permanent staff employed by the IO	Number of employees
Finances	Amount of financial resources provided by different sources	US$

To measure IOs’ de jure financial capabilities, we use a five‐level additive index. This indicator assesses the financial resources the agent is allowed to tap. De jure financial capabilities are weakest (‘1’) when the agent is dependent solely on voluntary member state contributions. When an agent is not allowed to access other financial resources, it cannot engage in autonomous action as every policy decision and even administrative expenses are subject to the principals’ veto. De jure financial capabilities increase with the number of additional financial resources. They are strongest (‘5’) when the agent has access to obligatory member state contributions, when the agent is allowed to charge fees for services, to accept voluntary contributions from non‐member states and private actors, and to levy taxes or collect similar mandatory contributions.^5^

The second set of indicators turns to IOs’ de facto capabilities. De facto staff capabilities are measured by the number of staff permanently employed at an IO.^6^ The higher this number is, the more capabilities an IO has to perform its tasks. To measure de facto financial capabilities, we turn to the agent's annual financial income. Corresponding to our de jure indicator, we include all available financial resources. The more financial resources are available to an IO, the higher is its capability to perform its tasks.

## The dataset: IO power and empowerment

To gauge IOE in terms of capabilities, we analyze six well‐known IOs. Following normal usage in the literature, we define an IO as a formal entity that has states as members and possesses a permanent secretariat or other indication of institutionalization, such a distinct physical location, a written constitution or convention, or a decision body that meets at least once a year (Pevehouse et al., 2004, p. 103). Based on this definition, Hooghe and Marks (2015) identify 72 IOs. From this set of organizations, we selected the EU, GATT/WTO, IMF, WHO, UNESCO, and WBG. We use this sample to illustrate that IOs with high or low tasks and issue scope have different capabilities that also shape their power. By adding our measures of IO capabilities, we provide a more complete picture of how the power of the selected IOs varies across IOs and over time. Only if we take into account the different configurations of tasks, issue scope, and capabilities, can we determine the power of IOs and its development over time. Consequently, the sample aims to represent different types of IOs as regards the other two principal components of IO power (task and issue scope).

First, we have selected IOs with different types and numbers of formally delegated tasks. The EU, IMF, and WBG perform a broad array of both regulatory and technical tasks, such as fulfilling executive functions, setting policy agendas, drafting budgets, and initiating member state accession and constitutional revision. According to Hooghe and Marks (2015), the EU has the highest score of formal delegation in their sample of 72 IOs, with the IMF ranking 9th and the WBG 25th. These three IOs are powerful in terms of the number and types of tasks they perform. By contrast, the UNESCO ranks 48th, the WHO 49th, and GATT/WTO 51st. These IOs are less powerful, performing only a limited number of tasks.

Second, the sample represents IOs with different levels of issue scope. Following Lenz et al. (2015), we distinguish general‐purpose and task‐specific IOs. General‐purpose IOs are powerful in terms of issue scope because they are involved in many issue areas, whereas task‐specific IOs are less powerful due to their focus on a narrow set of issue areas. The EU represents the set of powerful, general‐purpose organizations. In fact, the EU has the broadest issue scope in the Hooghe and Marks dataset (24 out of 25 possible issue areas). The remaining IOs in our sample represent task‐specific organizations from different issue areas. The GATT/WTO and the IMF deal with trade and financial issues. The UNESCO is active in cultural issues. The WHO focuses on health and the WBG on development.

The dataset measures IO capabilities annually from the founding of the selected IOs through 2015. For each IO‐year, we measure de jure and de facto staff and financial capabilities. As regards the de jure dimension, our focus is on formal rules set by treaties, constitutions, conventions, statutes, and rules of procedure. Data on the de facto dimension were gathered from published IO annual reports, financial statements, direct queries to the Human Resources Departments of IOs, and archival resources. The next section presents our findings regarding de jure and de facto financial capabilities. We subsequently discuss the results on de jure and de facto staff capabilities.

### Financial capabilities

How do the financial capabilities of the selected IOs evolve over time? De jure financial capabilities of IOs are strong when agents are allowed to tap diverse sources. De facto financial capabilities are weak when the income generated from these sources is low.

The organizational structures of the GATT/WTO, IMF, WHO, and UNESCO are relatively simple. The EU and WBG have more complex structures, uniting a diverse set of organizational bodies. For the EU, we have collected data on the Council of the EU, the European Commission, the European Parliament, and the European Court of Justice. For the WBG, our data includes information on the International Bank for Reconstruction and Development (IBRD), the International Development Association (IDA), the International Finance Corporation (IFC), the Multilateral Investment Agency (MIGA), and the International Centre for Settlement of Investment Disputes (ICSID). For the sake of parsimony and comparability, we report the data for the overall de jure and de facto financial capabilities of both organizations. De jure capabilities for the EU and the WBG are presented as mean values. Data on de facto finances reflect the sum of income for all sub‐bodies. These numbers are given in millions of constant US$.^7^

**Figure 1:**
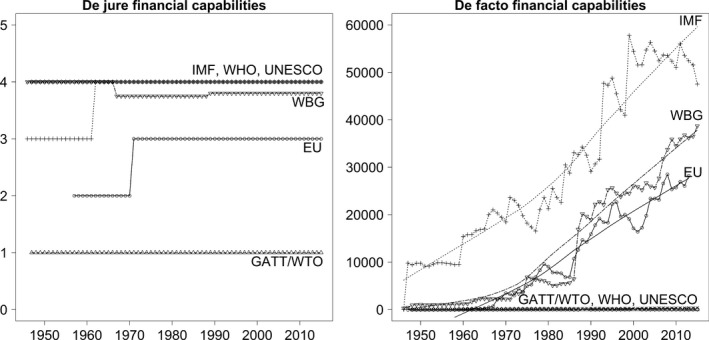
Financial capabilities across IOs and over time.

The left‐hand plot in Figure 1 depicts de jure financial capabilities. They remain essentially constant for all six IOs in the sample. We note invariably high financial capabilities for the WHO and UNESCO. Both IOs have not only formal access to member states’ voluntary contributions but are also allowed to tap obligatory member assessments, to charge fees for services, and to accept voluntary contributions from non‐members. For the IMF, the index score increases from three to four in 1962, when the General Agreement to Borrow gave the IMF the right to supplement the quota resources provided by member states by borrowing additional currencies. The slight variation for the WBG is the result of different rules for different sub‐bodies. IBRD, IDA, IFC, and MIGA are allowed to generate financial income from many sources, excluding only taxes and other mandatory contributions. ICSID's rules are more restricted as it is forbidden from accepting voluntary third party contributions. The increase for European institutions results from the creation of the EU as a customs union. Since, 1971, the EU collects, in addition to mandatory member contributions, import duties, fines, and fees for services from non‐EU countries. The GATT/WTO has the lowest de jure financial capabilities as it is only allowed to generate additional income from rental fees and publications.

Overall, our data display a remarkably stable ranking. In contrast to Graham's (2016) findings on the transformation of funding rules of United Nations programs, the financing rules of the IOs analyzed here appear to be robust to change. Despite diverging member state preferences over these IOs’ tasks and issue scope and a growing number of member states, we do not observe an increase in de jure financial capabilities. This ranking of the IOs in our sample does not correspond to the number of delegated tasks. Rather, we see that two organizations with a relatively limited set of tasks – the WHO and UNESCO – are given extensive rights to generate income, whereas the EU, which performs a greater number of tasks, is more restricted. In contrast, both the number of tasks and de jure financial capabilities are low for the WTO. Finally, the IMF and the WBG are equipped with strong de jure financial capabilities. This result may be explained by their respective issue areas. As the IMF and the WBG are tasked with providing financial assistance to member states, giving them strong de jure financial capabilities is an obvious solution to help them generate the resources they require.

**Figure 2:**
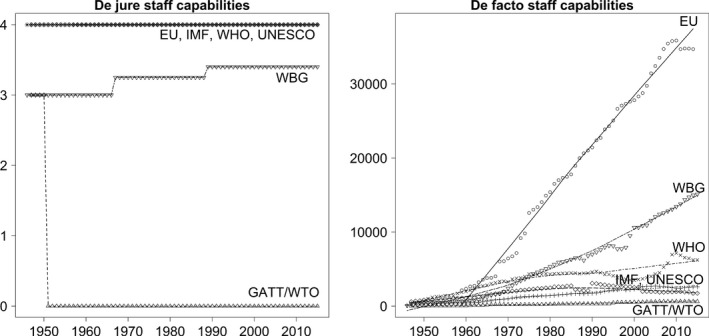
Staff capabilities across IOs and over time.

Do these rules shape de facto financial capabilities? What levels of financial revenue can we observe, and how do they change over time? The right‐hand plot in Figure 2 shows our results. Three main observations stand out. First, in line with the EU's relatively limited de jure financial capabilities, its de facto financial capabilities grow less strongly than the IMF's and the WBG's revenue. This is even more remarkable when we take into account the more limited tasks and scope of the latter two IOs. Yet, it has to be noted that IMF and WBG income is subject to tight lending rules. In contrast to the EU's annual revenue, these resources cannot be spent in full. Financial IOE seems to be less shaped by initial income levels than by task and scope expansion. With a total income of 12 million constant US$ in 1958, the EU is ranked at the lower bound of the distribution. Only GATT/WTO, which was then a regime without an organizational structure, has a lower income at this point in time (560,000 constant US$). The WBG was able to generate much higher levels of income – 467 million constant US$ in 1947 – during its early years. By, 1958, this amount had grown to 1.1 billion constant US$. The IO with the highest level of initial income was the IMF at 9.8 billion constant US$ in 1947. Due to stable quotas, this level remained almost constant until, 1959.

Second, over time, this ranking changes considerably. Today, the EU's income of more than 28.2 billion constant US$ is much higher than the WTO's (29 million constant US$), the WHO's (349 million constant US$), and UNESCO's (84 million constant US$). Only the financial capabilities of the IMF (47.5 billion constant US$) and the WBG (38.7 billion constant US$) are stronger. These trajectories over time do not seem to be shaped by the organizations’ de jure financial capabilities. Rather, we note that the ranking corresponds more strongly to the number of delegated tasks and to the issue scope and area. The three IOs for which we observe substantial growth in income (EU, IMF, and WBG) rank high in reference to the numbers of tasks and they perform these tasks – at least partly – in economic and financial issue areas.

Finally, although annual changes vary considerably across the three high‐income IOs, in the long run, their income appears to have been shaped by a similar general trend. The smooth functions for the EU, IMF, and WBG indicate that the income of all three organizations is characterized by continuous growth. Despite some dips and varying slopes, we observe a gradual empowerment of these organizations.

Overall, we draw two conclusions. First, financial capabilities vary considerably across organizations and over time. The IMF and the WBG are equipped with high de jure financial capabilities, and they are able to generate substantial income. Their financial capabilities are high on both dimensions. A medium level of financial capabilities can be noted for the EU, UNESCO, and WHO. Although the EU's de jure financial capabilities are lower, it is able to generate substantial income. The UNESCO and WHO are characterized by the reverse constellation. Both organizations are formally allowed to receive income from a variety of sources but they generate less income than the EU. Nonetheless, more recently, the WHO has been particularly successful in attracting external funding, for instance, by the Bill and Melinda Gates Foundation. With 277 million constant US$ of external funding, the WHO generated almost 80 per cent of its overall income from external sources in 2015.^8^ Yet, compared to the high‐income organizations in the sample their financial capabilities remain low over the entire observation period. GATT/WTO's financial capabilities have been low since its foundation. Institutionally restricted to small income from fees for services and member state contributions, the organization's success in generating income has been marginal.

Second, the trajectory of de facto financial capabilities does not seem to have been driven by the respective rules. Despite institutional limitations, the EU was able to expand its income considerably, whereas less restricted organizations, such as the UNESCO and WHO, were less successful in generating income. These differences in de facto financial capabilities might be driven by the expansion of IOs’ tasks and scope.

### Staff capabilities

How do the staff capabilities of the selected IOs evolve over time? The de jure staff capabilities of IOs are weak when principals have mechanisms at their disposal to interfere in staff selection. De facto staff capabilities are strong when the number of permanent staff is high.

As in the previous section on financial capabilities, we report the results for staff capabilities over time. The data are presented as total values for all organizations. The score for de jure staff capabilities is presented as the mean value of scores for all sub‐bodies. The reported number of permanently employed staff represents the sum of all staff employed at sub‐bodies.

The left‐hand plot in Figure 2 shows the development of de jure staff capabilities. Given the considerable variation in the form and function of the selected IOs, we observe surprisingly little variation across IOs and over time. For the EU, IMF, WHO, and UNESCO, de jure staff capabilities are invariably high. Since their creation, all four organizations have had the right to recruit staff without explicit principal approval. The slight variation for the WBG results from the fact that some of its sub‐bodies require collective principal approval while others do not. The IBRD, IDA, and IFC are required to recruit staff under the direction of the executive board, whereas the MIGA and ICSID are not. As ICSID was founded in 1966 and MIGA in 1988, the mean score for the WBG increases in these years. A different pattern is observable for GATT/WTO. The early GATT secretariat was required to recruit staff under the supervision of the collective principal. A revision of the GATT in 1951 resulted in the removal of this rule. Since then, neither GATT's constitutional rules nor the agreement establishing the WTO specify rules for staff recruitment. The organization's self‐description as a member‐driven organization where all decisions are made by member governments, suggests that there is nonetheless little institutional leeway in terms staffing decisions. Our sample includes five organizations with high de jure staff capabilities and one organization with low de jure staff capabilities. This result seems to correspond roughly to the number of tasks delegated to these IO.

Do these rules shape the number of permanent IO staff? What levels of de facto staff capabilities can be observed and how do they change over time? The right‐hand plot in Figure 2 depicts our results. It shows considerably more variation than our data on de jure staff capabilities. The IOs in the sample had substantially different numbers of staff at the time of their founding. At the lower bound of the distribution, we find the GATT/WTO, which had a small secretariat of only ten staff members. Because the creation of an International Trade Organization had failed, GATT was initially merely an international regime without an international bureaucracy. Thus, these low numbers are not very surprising. Nonetheless, the other two major international financial organizations, which were created at the Bretton Woods conference in 1944, were not equipped with a considerably larger staff. The WBG started operations with 72 permanent staff members and the IMF with just 100 permanent professionals. By contrast, the WHO was founded in 1948 with an almost three times larger permanent staff of 259 and UNESCO was created in 1945 with a permanent staff of 600. While these numbers point already to substantial differences during the founding years of the IOs in the sample, the 2,141 permanent staff working at the EU's predecessor organization – the European Coal and Steel Community – illustrate an even more significant variation, with a level of de facto staff capabilities well above that of the other five organizations.

Turning to changes over time, we note a gradual empowerment of all IOs in the sample. The data indicate, first, that de facto staff capabilities have grown. Second, the data suggest that higher initial levels of permanent staff translate into higher levels of permanent staff over time. European institutions were equipped with the largest permanent staff of all IOs examined at the time of their creation. With 34,705 permanent professionals employed at the end of the observation period, the EU's staff is still considerably larger than for the remaining IOs in the sample. Yet, the trajectories of these organizations are characterized by quite different slopes. This effect is most pronounced for the WBG, where permanent staff levels during the founding period with only 150 officials were almost 30 times lower than for the EU. This relative gulf between both organizations has narrowed substantially over time. With a permanent staff of more than 15,000, today's WBG is equipped with the second largest staff in the sample. It is now almost half the size of the EU. Similarly, the ranking of UNESCO has changed considerably over time. While UNESCO had the second largest permanent staff of the sample at its foundation, it fell over time to rank five due to slow staff growth, reaching only 1,734 by, 2015. Although staff developments at the GATT/WTO, IMF, and WHO show different trajectories over time, their ranking in the sample has remained constant. With 645 permanent professionals, GATT/WTO still has the smallest staff. The IMF remains fourth ranked in the sample, now employing a permanent staff of 2,611. The WHO is still ranked third, with a permanent staff of 6,233.

Overall, two results stand out. First, staff capabilities of IOs vary considerably across IOs. While the staff capabilities of the EU, an IO performing many tasks in a broad array of issue areas, have been high and growing since its creation, the staff capabilities of the WBG, an IO with more limited tasks and scope, has caught up over time. A medium level of staff capabilities can be noted for the IMF, WHO, and UNESCO, where formal rules provide for strong staff capabilities, whereas the number of staff is considerably smaller than for the EU and WBG. Low staff capabilities can be noted for GATT/WTO. Here, no formal rules on staffing exist for most of the observation period. Also, the number of permanent staff is still low despite the vast expansion of trade issues regulated by the WTO and the growing complexity of its trade negotiations.

Second, despite relatively similar formal rules, the temporal dynamics shaping the growth of permanent staff levels differ considerably across IOs. For the EU, our data indicate almost linear growth rates for the entire observation period. For the WBG, the slope is much flatter during the 1960s and 1970s and only becomes steeper at the end of the 1970s, when the number of projects financed by the Bank began to increase. By contrast, growth rates are much smaller for GATT/WTO. The IMF, UNESCO, and WHO even experience decreasing numbers of permanent staff during the 2000s.

## Conclusion

We have argued in this contribution that IO power can be measured on the basis of three principal components: tasks, issue scope, and capabilities. These components are subject to both formal and informal process of IOE over time. To illustrate how IO power changes both formally and informally, we have introduced a novel dataset which not only measures de jure but also de facto capabilities. These capabilities have two dimensions: staff and finances. IO power is higher when an IO has more permanent staff working to accomplish its goals and the more financial resources available to an IO, the higher is its power.

Our data demonstrate that IO capabilities vary across IOs and over time. By integrating our empirical observations with data on the selected IOs’ tasks and issue areas it becomes possible to compare power across IOs and over time. The only IO in the sample for which we diagnose both strong staff and financial capabilities is the WBG. Given the WBG's expansion of tasks and scope, it seems reasonable to argue that it has become more powerful over time. By contrast, the only IO in the sample with weak capabilities is GATT/WTO. Here, both staff and financial capabilities are low. Taking into account the GATT/WTO's expansion of tasks and scope, it seems that, today, the WTO has fewer capabilities for performing a broader array of more complex tasks. Consequently, we consider the WTO to have become less powerful in individual tasks and issue areas. The remaining organizations in the sample are characterized by a medium level of capabilities. While the EU's staff capabilities are high, its financial capabilities remain at a medium level. In combination with the strong expansion of tasks and scope, this result reflects a clear increase of power over time. Conversely, the IMF has strong financial capabilities but is more restricted in regards to staff. Taking into account the IMF's gradual expansion of scope to include issues of development, we also note empowerment in relation to the IMF's tasks. For the UNESCO and WHO, we find medium levels of both types of capabilities. Over time, the overall power of both IOs appears to have remained rather constant. Neither tasks and scope nor capabilities have grown significantly since their creation. These results are confirmed by Bauer and Ege's (2016) analysis of the bureaucratic autonomy of IO secretariats. Building on a broader set of indicators not only addressing IO power but also IO autonomy, the authors’ data indicate a similar ranking of IOs. Overall, our findings illustrate that IO tasks, issue scope, and capabilities constitute three components of IO power which may grow in parallel – as in the case of the EU – or which may develop in different directions – as in the WTO case. While we do not intend to generalize this finding on the relationships between components of IO power to the larger population of IOs, our cases reveal that different configurations of tasks, issue scope, and capabilities exist and result in different levels of power both across IOs and over time.

Based on these results, further research could focus on explaining the observed variance. Why do capabilities vary across IOs and over time? What drives general trends of IOE? How do different components of IO power develop over time and what shapes their configuration (see Conceição‐Heldt and Schmidtke, 2017; Graham, 2016 for first steps in this direction)? Our results permit a few conjectures. First, there appears to be a link between IO capabilities and tasks. The IOs in our sample with growing numbers of formal tasks are also characterized by growing capabilities. Second, there seems to be a relationship between financial capabilities and issue scope. For those IOs active in economic and financial issue areas, such as the EU, the IMF, and the WBG, financial capabilities are higher overall and grow more strongly over time than for IOs active in other issue areas. These potential relationships notwithstanding, the case of the WTO for which we note growing tasks and issue scope but stagnating capabilities suggests that capabilities do not follow directly from the other two components of IO power. Consequently, taking into account capabilities as a third component helps to identify such cases. Finally, turning more directly to the temporal dynamics of IO capabilities, we conjecture that initial levels of staff and financial capabilities may shape path dependent processes of IOE over time. Specifically, we expect that IOs with high initial levels of staff capabilities should be characterized by stronger IOE because this initial staff will use its resources to expand both staff and financial capabilities. When staff attempts to maximize resources occur within a favorable institutional context – for instance, low levels of principals’ attentiveness and cohesiveness, low effectiveness of principals’ control mechanisms, or a high level of agenda setting authority for agents – the likelihood of IOE might increase over time.

## Notes

1 Barnett and Finnemore (2004, p. 30) call this the regulatory and constitutive effects of power.

2 These processes are not unidirectional and IOs may also be disempowered over time.

3 We limit this indicator to two thirds of staff or more because, in most IOs, the recruitment of top level management staff is invariably subject to principal approval. Hence, no variance below this level could be captured by examining approval requirements for all staff.

4 More nuanced studies on IO staff recruitment also analyze more specific selection criteria such as geographic representation or meritocracy (see, for instance, Bauer and Ege 2012; Parizek 2016).

5 Note that our measure of capabilities is indifferent to the issue of IO autonomy. As discussed by Ege and Bauer (2017) and Graham (2017) different financial sources may limit IO autonomy.

6 Recent research has begun to distinguish different categories of IO staff, such as professional staff, which is mainly involved in policy‐related work, and service staff, which provides administrative support (see, for instance, Ege and Bauer, 2017). We include both groups because they contribute – albeit in different ways – to staff capabilities.

7 To adjust the raw data in nominal US$, we divide it by the Consumer Price Index provided by the US Bureau of Labor Statistics (2016).

8 For a discussion of the potential effects of this changing mix of financial sources see Ege and Bauer (2017) and Graham (2017).
